# Supplementary material for: Tubulin autoregulation mediator TTC5 regulates neuronal morphology and migration
Source: bioRxiv. 2026 Jun 24:2026.06.23.733857. Preprint. [Version 1] doi: 10.64898/2026.06.23.733857 (PMC13370610; doi:10.64898/2026.06.23.733857)
Supplement: Supplement 1 [file NIHPP2026.06.23.733857v1-supplement-1.pdf]

bioRxiv preprint doi: https://doi.org/10.64898/2026.06.23.733857; this version posted June 24, 2026. The copyright holder for this preprint (which was not certified by peer review) is the author/funder. This article is a US Government work. It is not subject to copyright under 17 USC 105 and is also made available for use under a CC0 license.

Supplemental Figures

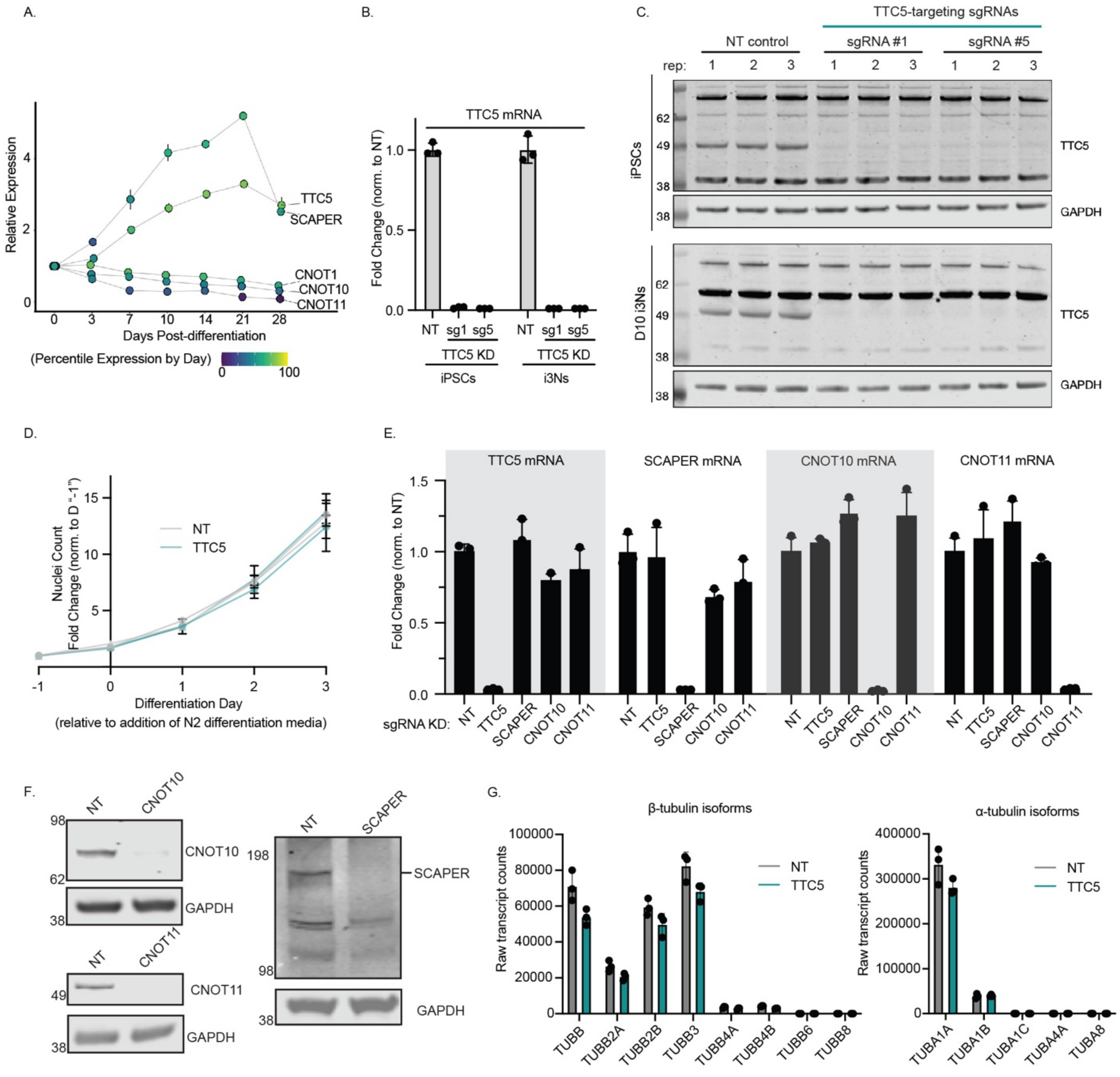

Figure S1. Baseline characterization and validation of tubulin autoregulation effector KDs from iPSCs to i^{3}Neurons (related to Figure 1).

(**A**) Relative protein expression levels of indicated genes across a timecourse of i3N differentiation[25] generated using https://niacard.shinyapps.io/i3Neuron/.

(**B - C**) Control and TTC5 KD iPSC lines were generated in parallel via lentiviral transduction of iPSCs with two independent TTC5 promoter-targeting guides or a non-targeting (NT) control followed by selection and differentiation into i3Ns. At iPSC stage or D10 post-differentiation, cells were harvested and assayed for TTC5 mRNA and protein expression by (**B**) RT-qPCR, normalized to HPRT and NT control and (**C**) Western blot with GAPDH as control. TTC5 sg5 was used for all subsequent TTC5 KD studies.

(**D**) Cell proliferation across early differentiation (iPSC to D3 i3N) quantified as number of Halo-NLS nuclei for NT (gray) and TTC5 KD (teal) cells; n = 10 wells/condition. Error bars, mean +/-SD; individual lines represent two independent experiments.

bioRxiv preprint doi: https://doi.org/10.64898/2026.06.23.733857; this version posted June 24, 2026. The copyright holder for this preprint (which was not certified by peer review) is the author/funder. This article is a US Government work. It is not subject to copyright under 17 USC 105 and is also made available for use under a CC0 license.

(**E - F**) KD efficiency for TTC5, SCAPER and CCR4-Not1 complex components CNOT10 and CNOT11 in D14 i^3Neurons at the (**E**) RNA level by RT-qPCR, normalized to HPRT and NT control, and at the (**F**) protein level by Western blot, with GAPDH control.
(**G**) Raw transcript counts for all expressed (> 0 count) α-and β-tubulin isoforms obtained from RNA-Seq of untreated NT and TTC5 KD D14 i3Ns shown in Figure 1D.

bioRxiv preprint doi: https://doi.org/10.64898/2026.06.23.733857; this version posted June 24, 2026. The copyright holder for this preprint (which was not certified by peer review) is the author/funder. This article is a US Government work. It is not subject to copyright under 17 USC 105 and is also made available for use under a CC0 license.

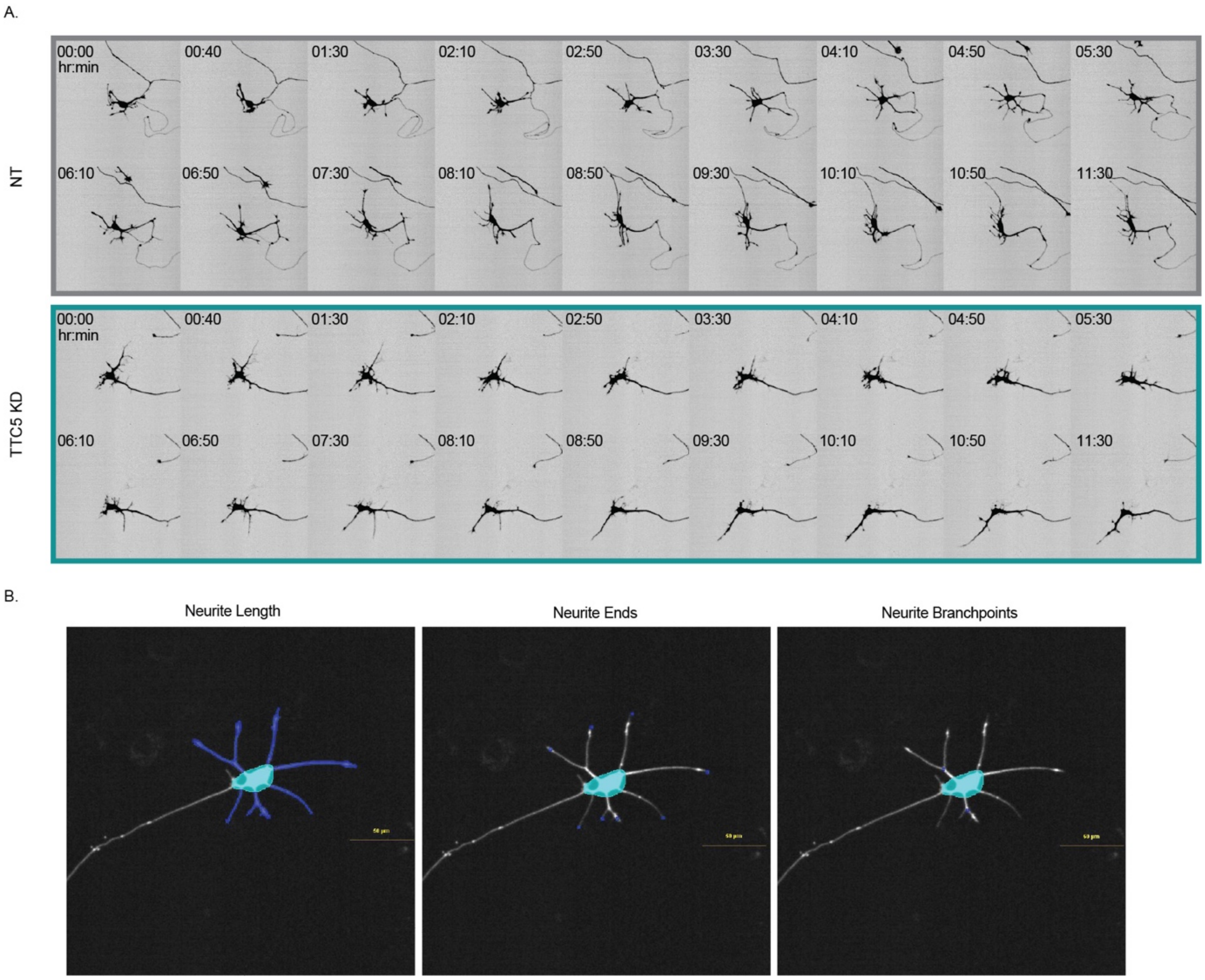

Figure S2. Representative examples of image collection and automated analysis of arborization metrics (related to Figure 2).

(**A**) Montage of frames (every fourth frame) of mScarlet-expressing D12 i3Ns for arborization analysis presented in Figure 2A illustrating dynamic extension and retraction of processes for individual neurons (also see Movie S1).

(**B**) Snapshots from customized Nikon GA3 automated pipeline (Methods) highlighting identification in dark blue, from left to right, of neurites (with omission of longest axonal process), neurite ends and neurite branchpoints.

bioRxiv preprint doi: https://doi.org/10.64898/2026.06.23.733857; this version posted June 24, 2026. The copyright holder for this preprint (which was not certified by peer review) is the author/funder. This article is a US Government work. It is not subject to copyright under 17 USC 105 and is also made available for use under a CC0 license.

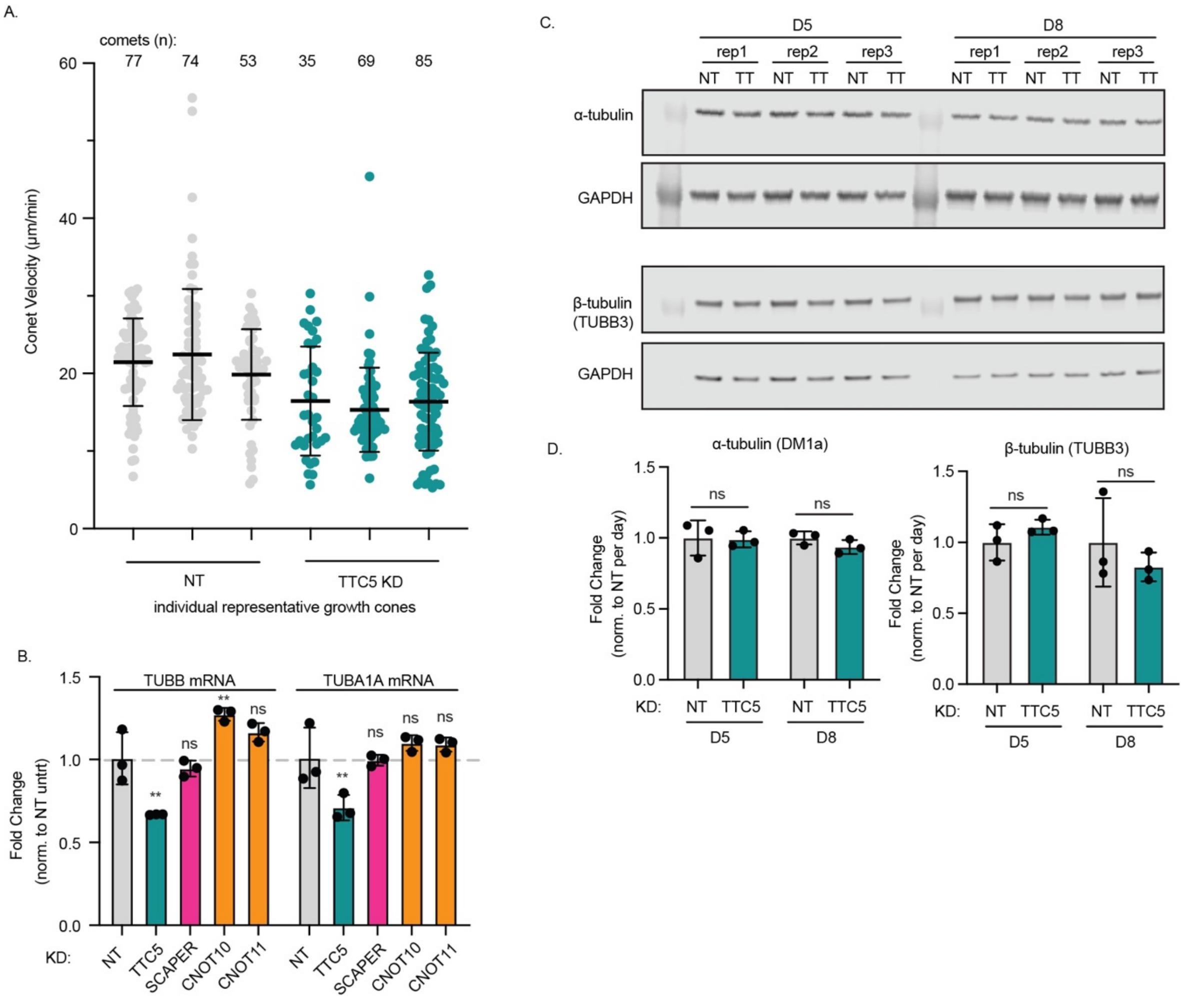

Figure S3. Additional characterization of EB1 comet dynamics and tubulin expression levels upon TTC5 KD (related to Figure 2).

(**A**) EB1-GFP comet velocities for three representative individual growth cones for NT and TTC5 KD used to obtain average comet velocities per growth cone in Figure 2F. Error bars, mean +/-SD.

(**B**) TUBB and TUBA1A mRNA levels assessed by RT-qPCR in D14 i3Ns upon KD of indicated gene normalized to NT control (following normalization to HPRT); error bars indicate mean +/- SD ; **, $p < 0.01$, by one-way ANOVA with Dunnett's multiple comparisons test.

(**C**) Western blot for α-tubulin and β-tubulin (TUBB3) tubulin protein in NT and TTC5 KD neurons at D5 and D8 in triplicate and quantified in (D) normalized to GAPDH; error bars indicate mean +/- SD; significance determined by unpaired t-test.

bioRxiv preprint doi: https://doi.org/10.64898/2026.06.23.733857; this version posted June 24, 2026. The copyright holder for this preprint (which was not certified by peer review) is the author/funder. This article is a US Government work. It is not subject to copyright under 17 USC 105 and is also made available for use under a CC0 license.

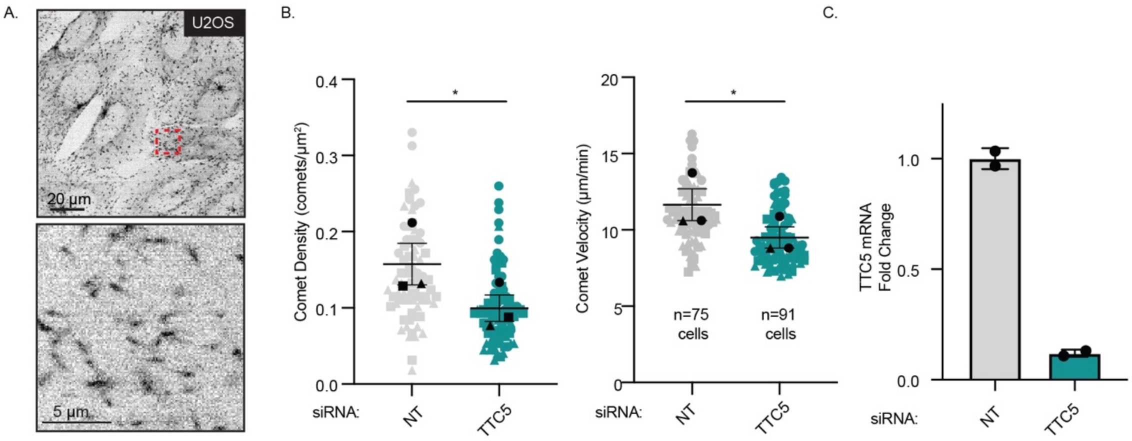

Figure S4. TTC5 loss results in reduced microtubule density and growth rate in U2OS cells (related to Figure 2).

(**A**) Representative frame from a movie of EB1-mNG knock-in U2OS cells (top; scale bar: 20 µm) and magnified region of an U2OS cell showing individual EB1-mNG comets (bottom; scale bar: 5 µm).

(**B**) EB1 comet density (left) and velocity (right) for NT (grey) or TTC5 siRNA KD (teal) cells. Individual points represent average values for individual cells from three independent experiments; n= 75, 91 cells for NT and TTC5 KD, respectively; error bars and symbols indicate the paired means of each independent experiment +/- SEM; *, $p < 0.05$ by paired t-test.

(**C**) TTC5 mRNA level upon siRNA KD in U2OS cells assessed by RT-qPCR normalized to NT control (following normalization to GAPDH).

bioRxiv preprint doi: https://doi.org/10.64898/2026.06.23.733857; this version posted June 24, 2026. The copyright holder for this preprint (which was not certified by peer review) is the author/funder. This article is a US Government work. It is not subject to copyright under 17 USC 105 and is also made available for use under a CC0 license.

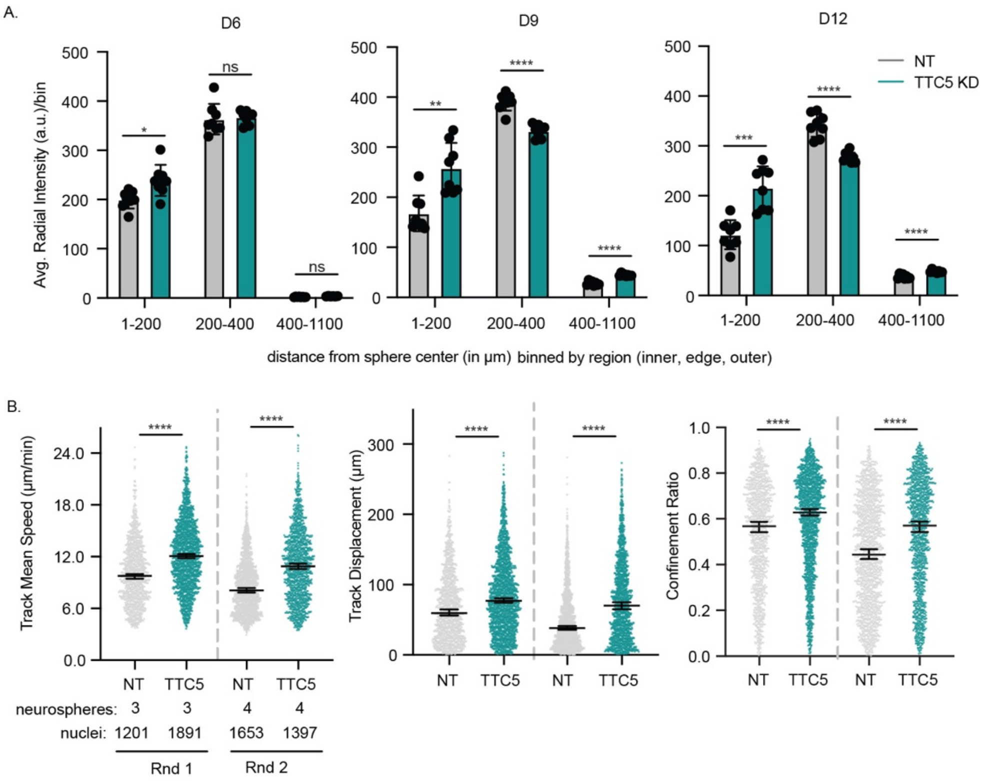

Figure S5. Alternate graphical depiction of static and dynamic motility readouts in Figure 3 (related to Figure 3).

(**A**) For each timepoint (D6, D9, D12), the average signal of dispersed nuclei was divided into bins based on relative distance from the center and edge of each neurosphere (as in Figure 3B, right panel). Statistical significance was determined for the average signal from each bin; corresponding with the continuous plots depicting nuclear signal intensity at distances from the nucleus displayed in Figure 3B. Error bars indicate mean Halo-tagged nuclear signal within each indicated bin +/- SD, statistical significance determined by unpaired t-test (NT= 8 neurospheres, TTC5 KD=8 neurospheres); *, $p<0.05$, **, $p < 0.01$, ***, $p < 0.001$, ****, $p < 0.0001$.

(**B**) Scatterplot of data represented as CDFs in Figure 3D of nuclear motility parameters for NT versus TTC5 KD neurospheres (Rnd 2 used for representative depictions in main figure). Error bars indicate the median with 95% confidence interval for parameters of individual nuclei pooled across multiple neurospheres for two independent experimental replicates; ****, $p < 0.0001$ by Kolmogorov-Smirnov test.

bioRxiv preprint doi: https://doi.org/10.64898/2026.06.23.733857; this version posted June 24, 2026. The copyright holder for this preprint (which was not certified by peer review) is the author/funder. This article is a US Government work. It is not subject to copyright under 17 USC 105 and is also made available for use under a CC0 license.

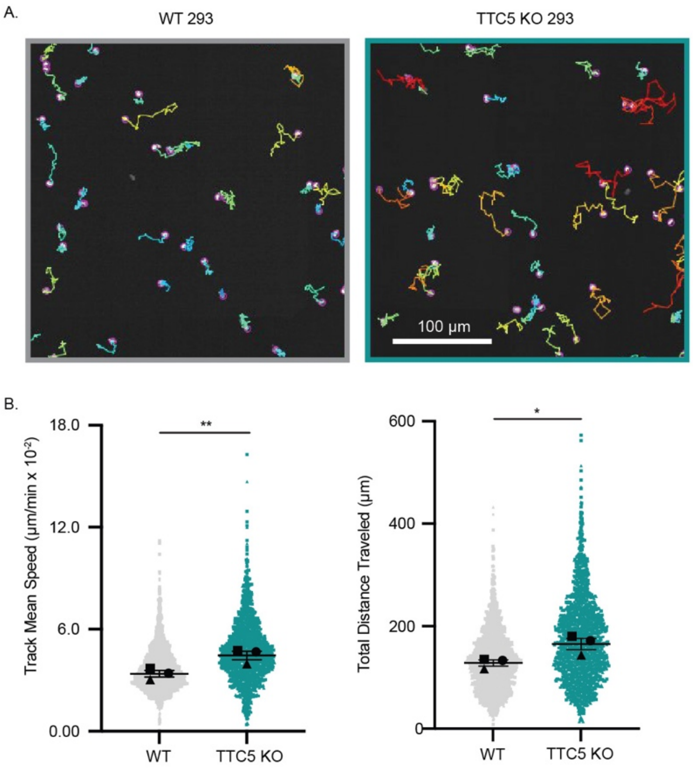

Figure S6. TTC5 loss results in altered motility in a non-neuronal cell line (related to Figure 3). (**A**)First frame from representative movie of Halo-tagged nuclei from WT and TTC5 KO HEK293 cells seeded onto fibronectin-coated plates (Methods). Nuclei are overlaid with tracks across a full eight-hour timecourse and color-coded by track mean speed from dark blue (0.00 mm/sec) to red (0.015 mm/sec).
(**B**) mean speed (left) and total distance traveled (right) of tracked Halo-NLS nuclei. Dots represent individual nuclei compiled across three independent experiments (n= 1855 nuclei for WT, 1724 nuclei for TTC5 KO) with error bars and symbol indicating the paired medians of each of these three independent experiments +/- SEM; *, $p < 0.05$, **, $p < 0.01$ by paired t-test.

bioRxiv preprint doi: https://doi.org/10.64898/2026.06.23.733857; this version posted June 24, 2026. The copyright holder for this preprint (which was not certified by peer review) is the author/funder. This article is a US Government work. It is not subject to copyright under 17 USC 105 and is also made available for use under a CC0 license.

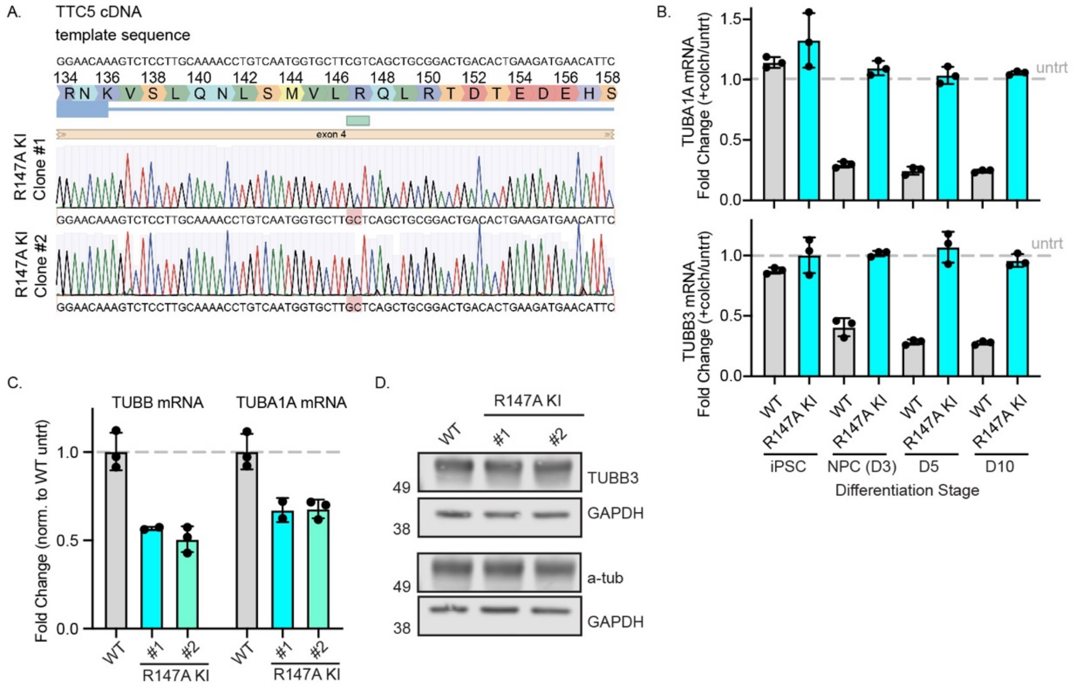

Figure S7. Line validation, tubulin autoregulation and tubulin level characterization in R147A KI lines (related to Figure 4).

(**A**) Sanger sequencing validation of R147A KI clones #1 and #2.

(**B**) Fold change for TUBB3 and TUBA1A mRNA level upon colchicine treatment relative to the untreated control for WT (gray) and R147A KI #1 (cyan) following initial normalization to housekeeping gene HPRT across differentiation from iPSCs to D10 i^{3}Neurons assayed by RT-qPCR. Each condition was assayed in triplicate.

(**C**) Fold change of tubulin mRNA levels relative to the WT control in untreated D14 WT and R147A KI i3Ns assayed by RT-qPCR as in (B).

(**D**) Quantification of tubulin protein levels by Western blot in untreated D14 WT and R147A KI i^{3}Neurons.

bioRxiv preprint doi: https://doi.org/10.64898/2026.06.23.733857; this version posted June 24, 2026. The copyright holder for this preprint (which was not certified by peer review) is the author/funder. This article is a US Government work. It is not subject to copyright under 17 USC 105 and is also made available for use under a CC0 license.

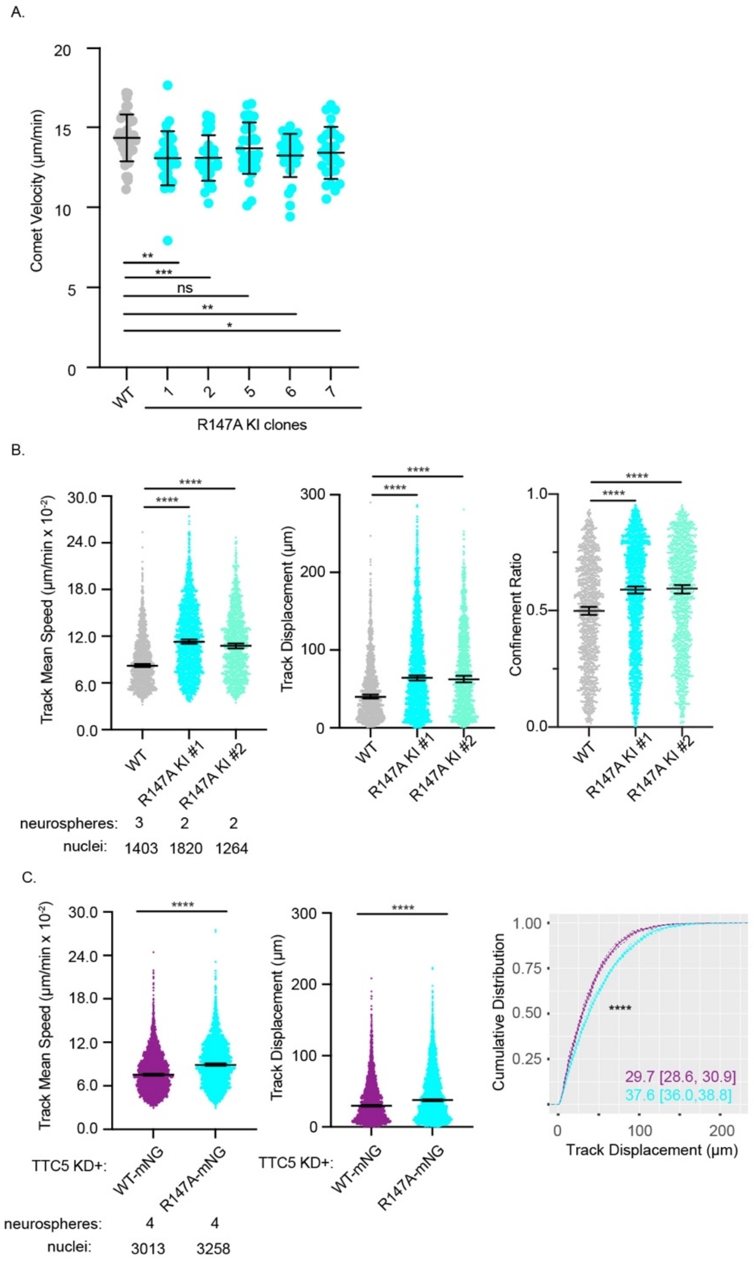

Figure S8. Additional characterization of neuronal phenotypes in the R147A knock-in and WT versus R147A mutant rescue i^{3}Neurons (related to Figure 4).

(**A**) Quantification of EB1-EGFP comet velocities across 5 independent R147A KI clones displaying mean +/-SD; *, $p < 0.05$, **, $p < 0.01$, ***, $p < 0.001$ by unpaired student's t-test.

(**B**) Scatterplot of data represented as a CDF in Figure 4H for nuclear motility parameters. Error bars indicate the median with 95% confidence interval for individual nuclei pooled across multiple neurospheres; ****, $p < 0.0001$ by Kruskal-Wallis test.

(**C**) Scatterplot of data represented as CDF in Figure 4L (track mean speed) or in rightmost panel (track displacement), of nuclear motility parameters for TTC5 KD neurospheres upon reintroduction of either WT (purple) or R147A (cyan) TTC5-mNG. Error bars indicate the median with 95% confidence interval for parameters of individual nuclei pooled across multiple neurospheres; ****, $p < 0.0001$ by Kolmogorov-Smirnov test.

bioRxiv preprint doi: https://doi.org/10.64898/2026.06.23.733857; this version posted June 24, 2026. The copyright holder for this preprint (which was not certified by peer review) is the author/funder. This article is a US Government work. It is not subject to copyright under 17 USC 105 and is also made available for use under a CC0 license.

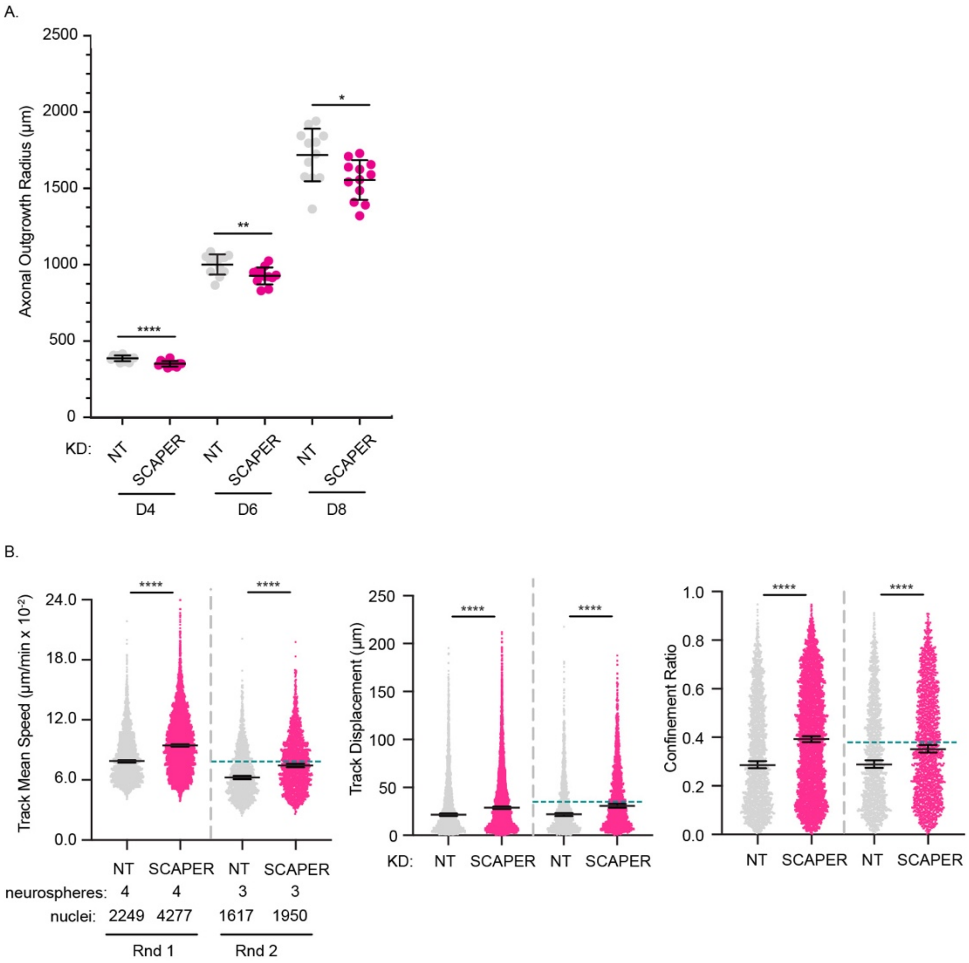

Figure S9. Additional neuronal assay characterization of SCAPER KD i^{3}Neurons (related to Figure 5).

(**A**) Independent experimental replicate of axonal outgrowth in NT versus SCAPER KD i3Ns assayed longitudinally at D4, D6 and D8 post-differentiation (n=12 spheres NT and 12 spheres SCAPER KD) as in Figure 5C. Error bars indicate the mean +/- SD with statistical significance determined by Welch's t-test; *, $p < 0.05$, **, $p < 0.01$, ****, $p < 0.0001$.

(**B**) Scatterplot of data shown as CDF in Figure 5D, tracking nuclear motility parameters. Error bars indicate the median with 95% confidence interval for parameters of individual nuclei pooled across multiple neurospheres for two independent experimental replicates, ****, $p<0.0001$ by Kruskal-Wallis test. Dotted teal line in Rnd 2 replicate indicates respective median value for TTC5 KD neurospheres (n = 2) collected side-by-side within the same experiment.

bioRxiv preprint doi: https://doi.org/10.64898/2026.06.23.733857; this version posted June 24, 2026. The copyright holder for this preprint (which was not certified by peer review) is the author/funder. This article is a US Government work. It is not subject to copyright under 17 USC 105 and is also made available for use under a CC0 license.

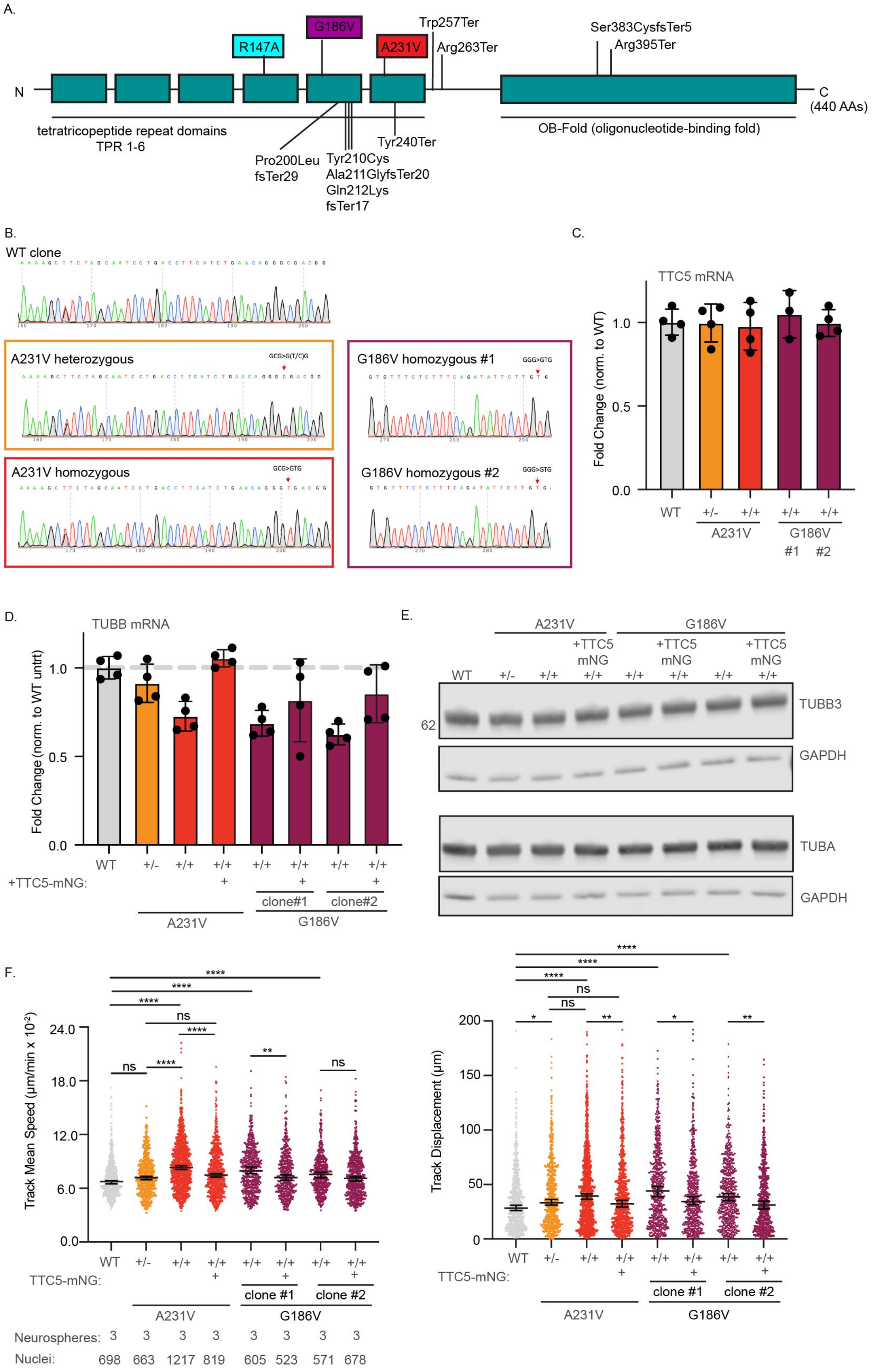

bioRxiv preprint doi: https://doi.org/10.64898/2026.06.23.733857; this version posted June 24, 2026. The copyright holder for this preprint (which was not certified by peer review) is the author/funder. This article is a US Government work. It is not subject to copyright under 17 USC 105 and is also made available for use under a CC0 license.

Figure S10. Baseline characterization and neuronal assay assessment for disease mutant KI lines (related to Figure 6).
(A) Schematic of reported disease mutants for TTC5 with KI mutants analyzed in this study highlighted alongside structure-guided mutant R147A (modified from Musante et al.[18]).
(B) Validation of disease mutant knock-ins via Sanger sequencing
(C) Validation of disease mutant TTC5 mRNA levels by RT-qPCR (normalized to WT clone following normalization to HPRT)
(D) Baseline TUBB mRNA level in untreated WT and disease mutant knock-in D10 i^3Neurons by RT-qPCR (normalized to WT clone following normalization to HPRT). Each condition was performed in duplicate across two independent experiments.
(E) Expression of α and β-tubulin protein in NT control and indicated disease mutant D10 i^3Neurons assayed by Western blot (with GAPDH control).
(F) Scatterplot depiction of data represented as CDF in Figure 6D (with inclusion of an additional G186V clone) of nuclear motility parameters track mean speed and track displacement. Error bars indicate the median with 95% confidence interval for individual nuclei pooled across neurospheres (n=3 per condition); *, $p < 0.05$, **, $p < 0.01$, ***, $p < 0.001$, ****, $p < 0.0001$ by Kruskal-Wallis test.

Supplemental Movies

Movie S1. Time lapse showing dendritic extension dynamics and automated analysis for NT and TTC5 KD i^3Neurons (related to Figure 2A, 2B).

Movie S2. Time lapse of EB1-GFP in NT and TTC5 KD i^3Neurons growth cones (related to Figure 2E,2F).

Movie S3. Time lapse of migration for NT and TTC5 KD i^3Neurons on glial bed (related to Figure 3C, 3D).

Movie S4. Time lapse of migration for WT and TTC5 R147A KI i^3Neurons on glial bed (related to Figure 4H).
